# Case Report: SMARCA4 (BRG1)-deficient undifferentiated carcinoma of gallbladder with genetic analysis

**DOI:** 10.3389/fonc.2023.1086266

**Published:** 2023-06-30

**Authors:** Xiangpeng Meng, Jia Ma, Nan Meng, Tianyu Yun, Beifang Niu

**Affiliations:** ^1^ Pancreatic Endocrinology Ward, Department of General Surgery, Shengjing Hospital of China Medical University, Shenyang, China; ^2^ Department of Gastroenterology, The Fourth Affiliated Hospital of China Medical University, Shenyang, China; ^3^ Beijing ChosenMed Clinical Laboratory Co. Ltd., Beijing, China; ^4^ Computer Network Information Center, Chinese Academy of Sciences, Beijing, China; ^5^ University of the Chinese Academy of Sciences, Beijing, China

**Keywords:** *SMARCA4*, *BRG1*, SWI/SNF, gallbladder carcinoma, case report

## Abstract

*SMARCA4 (BRG1)*-deficient undifferentiated carcinoma is a rare and highly aggressive malignancy. It has been reported to occur in a multiple range of organs. However, to the best of our knowledge, *SMARCA4 (BRG1)*-deficient undifferentiated carcinoma of gallbladder has not yet been reported. Here, we describe a case of *SMARCA4 (BRG1)*-deficient undifferentiated carcinoma of gallbladder. Through comprehensive genetic analysis, we hypothesized that in addition to *SMARCA4 (BRG1)* deficiency, other genetic changes might also be involved in the tumorigenesis of undifferentiated gallbladder cancer in this patient, particularly somatic mutations in the *CTNNB1, KRAS, PIK3CA, TP53, CREBBP*, and *FANCI* genes. To the best of our knowledge, this is the first report of *SMARCA4 (BRG1)*-deficient undifferentiated carcinoma of gallbladder with genetic analysis.

## Background

Gallbladder carcinoma (GBC) is a rare malignancy. Nearly 98% of GBCs are adenocarcinomas. Other rare histological types include mucinous, squamous, adenosquamous, small-cell, and undifferentiated carcinomas (1). Undifferentiated carcinoma of gallbladder accounts for 3.4% of all GBCs. This malignancy is more aggressive and frequently presents a larger tumor size compared to GBC (5.0 cm versus 3.0 cm). Its prognosis is extremely poor, with a median overall survival of 7.3 months.

The switch/sucrose non-fermenting (SWI/SNF) complex is a family of ATP-dependent chromatin remodeling proteins that play a crucial role in regulating gene transcription. SWI/SNF complex consists of 12–15 subunits encoded by 29 genes, including *SMARCA2 (BRM), SMARCA4 (BRG1), SMARCC1, SMARCC2, SMARCB1 (INI1), ARID1A*, and *PBRM1*. SWI/SNF complex exerts a crucial role in the differentiation, cell adhesion, motility of cancer cells and induces abnormal activation of the hedgehog signaling pathway (2).

*SMARCA4 (BRG1)* is one of the catalytic subunits of SWI/SNF complex. Inactivation of *SMARCA4 (BRG1)* has recently been suggested to be involved in the pathogenesis of some undifferentiated carcinomas. Previous studies have reported that *SMARCA4 (BRG1)*-deficient undifferentiated carcinomas could occur in the lung, ovary, gastrointestinal tract, uterus, and other organs (3–9). However, to the best of our knowledge, *SMARCA4 (BRG1)*-deficient undifferentiated carcinoma of gallbladder has not yet been reported.

Here, we report the first case of *SMARCA4 (BRG1)*-deficient undifferentiated carcinoma of gallbladder and analyze comprehensively its genetic alterations. We also discussed the potential targeted therapy.

## Case presentation

**Figure 1:**
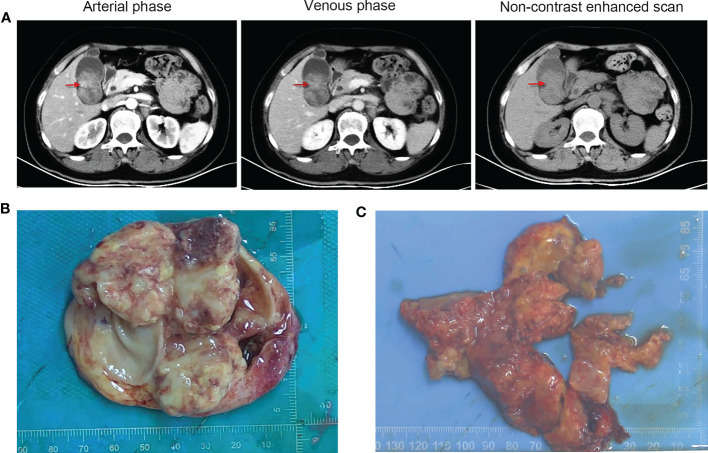
Radiographic and macroscopic images of the tumor. **(A)** Computed tomography images of the patient (red arrows refer to the space-occupying lesion). **(B)** The resected gallbladder was approximately 5.0 cm in diameter. **(C)** The irregular mass in the gallbladder was approximately 4.5 cm × 3.0 cm × 3.0 cm in size.

**Figure 2:**
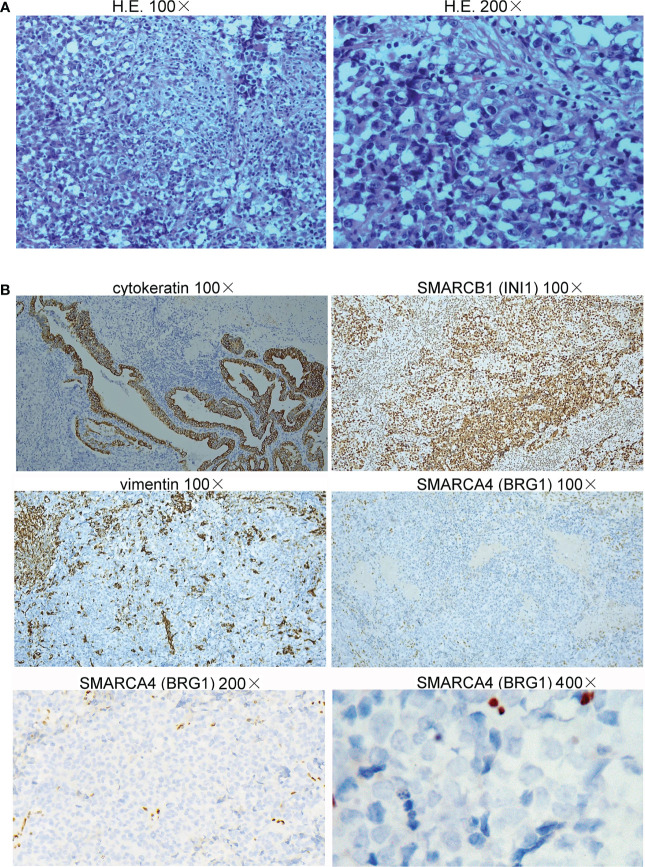
Histopathology of the undifferentiated carcinoma of the gallbladder. **(A)** The tumor cells presented obvious atypia, diffuse patchy infiltration, growth, and poor cell adhesion (H&E staining, 100× and 200×). **(B)** Immunohistochemical staining showed that the neoplastic cells were positive for cytokeratin and SMARCB1 (INI1) but negative for vimentin (100×) and SMARCA4 (BRG1) (100×, 200×, and 400×).

On June 30, 2022, a 65-year-old female patient was admitted to the hospital due to right upper abdominal pain for more than one month and aggravation for half a month. A whole abdominal enhanced computer tomography (CT) scan showed a space-occupying lesion in the gallbladder, with enlargement of multiple peripheral and retroperitoneal lymph nodes, suspected a malignant lesion (**Figure 1A**). On July 5, 2022, the patient underwent radical resection of the gallbladder, which was approximately 5.0 cm in diameter (**Figure 1B**). Upon partially opening the gallbladder, it was discovered that a mass took up a significant portion of it. The mass exhibited a yellow-white color and had a brittle texture upon being cut. Further inspection revealed that it had infiltrated the gallbladder’s muscle layer. The space-occupying lesion was approximately 4.5 cm × 3.0 cm × 3.0 cm in size (**Figure 1C**). Postoperative pathology revealed that the tumor cells presented obvious atypia, diffuse patchy infiltration, growth, poor cell adhesion, chromatin vacuolated, nucleoli obvious, mitotic figures were easy to see, necrosis and more lymphocyte infiltration were seen (**Figure 2A**). Notably, there were no cancer cells detected in the stump of the cystic duct, but retroperitoneal lymph node metastasis was observed. Immunohistochemistry (IHC) showed that the tumor cells were positive for cytokeratin, synaptophysin, SMARCB1 (INI1), and negative for SMARCA4 (BRG1), CK7, CK19, p63, p40, vimentin (**Figure 2B**). IHC for PD-L1 (DAKO 22C3) showed that the tumor proportion score (TPS) was <1%, and the combined positive score (CPS) was 50. The tumor had metastasized to the retroperitoneal lymph nodes as well as lymph node groups 7, 8, 9, 12, and 13. The final diagnosis was *SMARCA4 (BRG1)*-deficient undifferentiated carcinoma of gallbladder (stage II-III).

On July 14, 2022, DNA and RNA were extracted from the resected formalin fixed and paraffin embedded tumor tissues of the patient, and were subjected to next-generation sequencing (NGS) using a 1123-gene panel and a 102-gene panel, respectively (ChosenMed Technology [Beijing] Co. Ltd, Beijing, China). The sequencing results using DNA samples revealed that the patient harbored somatic mutations in the *CTNNB1, KRAS, PIK3CA, TP53, CREBBP, ERCC5, FANCI, FANCM, FAP* and *PTPRT* genes. The sequencing results using RNA samples revealed that the patient harbored four gene fusions: *LINC01138-NOTCH2, YWHAE-CRK, IGF2BP2-ETV5*, and *IGF2BP2-ETV5*. In addition, analysis of the NGS data revealed microsatellite-stability (MSS) status and medium tumor mutational burden (M-TMB, 8.22 muts/Mb). The genomic profile of the patient is demonstrated in **Table 1**. The patient had no obvious discomfort after the operation and was discharged from the hospital one month later. The patient died five months after surgery on December 14, 2022.

**Table 1:** Genomic profile of somatic alterations for the patient.

Sequencing results using DNA samples										
Gene	Transcript	Exon	Nucleotide	Amino acid	Mutant allele frequency	OncoKB annotation	MutationTaster prediction	SIFT prediction	Polyphen-2 prediction	Variant type
*CTNNB1*	NM_001904	3	c.109T>C	p.S37P	30.68%	Likely oncogenic				II
*KRAS*	NM_004985	2	c.35G>C	p.G12A	29.66%	Oncogenic				II
*PIK3CA*	NM_006218	21	c.3129G>T	p.M1043I	26.14%	Oncogenic				II
*TP53*	NM_000546	5	c.455C>T	p.P152L	33.33%	Likely oncogenic				II
*CREBBP*	NM_004380	31	c.5488G>A	p.A1830T	25.80%		Disease causing	Deleterious (score: 0)	Probably damaging (score: 0.999)	III
*ERCC5*	NM_000123	15	c.3281C>T	p.S1094L	26.69%		Disease causing	Tolerated (score: 0.11)	Possibly damaging (score: 0.953)	III
*FANCI*	NM_001113378	22	c.2177_2179del	p.A727del	8.00%		Disease causing	Not applicable	Not applicable	III
*FANCM*	NM_020937	14	c.2635G>T	p.D879Y	9.66%		Polymorphism	Deleterious (score: 0.03)	Possibly damaging (score: 0.855)	III
*FAP*	NM_001291807	8	c.598G>A	p.E200K	21.54%		Disease causing,	Tolerant (score: 0.27)	Possibly damaging (score: 0.665)	III
*PTPRT*	NM_133170	8	c.1382G>A	p.R461Q	19.18%		Disease causing	Tolerant (score: 0.21)	Probably damaging (score: 0.988)	III
Sequencing results using RNA samples										
Gene Fusion	Chromosome	Exon	Nucleotide	Amino acid		Variant type				
*LINC01138-NOTCH2*	LINC01138: NR_027468	1	LINC01138 (exon1)-NOTCH2 (exon2)			III				
	NOTCH2: NM_024408	2								
*YWHAE-CRK*	YWHAE: NM_006761	1	YWHAE (exon1)-CRK (exon2)			III				
	CRK: NM_016823	2								
*IGF2BP2-ETV5*	IGF2BP2: NM_001007225	2	IGF2BP2 (exon2)-ETV5 (exon6)			III				
	ETV5: NM_004454	6								
*IGF2BP2-ETV5*	IGF2BP2: NM_001007225	1	IGF2BP2 (exon1)-ETV5 (exon13)			III				
	ETV5: NM_004454	13								

SIFT Deleterious: score<0.05, Tolerated: score≥0.05.

Polyphen-2 Probably damaging: score ≥ 0.957, Possibly damaging: 0.453 ≤ score ≤ 0.956), Benign: score ≤ 0.452.

Variant type II: Gene variants that have potential clinical significance in terms of treatment, prognosis, or diagnosis; III: Gene variants with unknown clinical significance in terms of treatment, prognosis, or diagnosis, or variants that have never been reported in any cancer.

## Discussion

Gallbladder cancer (GBC) is an uncommon but highly fatal malignancy, and undifferentiated GBC is extremely rare. Some research had revealed the genomics profile of GBC. The common somatic mutation genes were *TP53* (64%-73%), *CDKN2A* (11.0%-25.0%), *ERBB2* (9.3%), and *PIK3CA* (10%-20%) in GBC (10, 11). The *SMARCA4* alteration frequency was 7.0% in a 60 GBC patients study (11). A case reported an SWI/SNF-deficient undifferentiated/rhabdoid carcinoma of the gallbladder carrying a POLE mutation in a 30-year-old woman (12).

The incidence of *SMRCA4* somatic mutations in NSCLC is 8% (407/4813); the major co-mutated genes were *TP53* (56%), *KEAP1* (41%), *STK11* (39%), and *KRAS* (36%) in SMARCA4-mutant NSCLC. Patients with *SMARCA4* co-mutations with *STK11* or *KEAP1* had a worse prognosis than those with single mutations, and patients with triple mutations had the worst prognosis (13).

In addition, SMARCA4 (BRG1)-deficient in other undifferentiated cancers has been reported sporadically, such as colon (14), gastrointestinal tract (15), and ovary (16). The SMARCA4 (BRG1)-deficient in undifferentiated gallbladder carcinoma has not yet been reported. This case first reported a patient with SMARCA4 (BRG1)-deficient undifferentiated gallbladder carcinoma. GBC is a multifactorial disease whose occurrence is related to chronic inflammation of the gallbladder, dietary factors and female gender. Multiple genetic alterations are involved in this malignancy, the most common being *KRAS, TP53*, and *erbB-2* (*neu*/*HER-2*) genes (17). In the patient, mutations in the *KRAS* (p.G12A) and *TP53* (p.P152L) genes were also detected, but no aberrations in *erbB-2*.

As one of the most prevalent mutations of *KRAS*, the mutation p.G12A in the *KRAS* has the potential capability to activate MEK/ERK- and PI3K/AKT-signaling pathways in cancer cells and is an oncogenic mutation (18).

*TP53* is a tumor suppressor in the DNA damage pathway. According to the annotation in the OncoKB database, *TP53* p.P152L may lead to loss of protein function and is probably oncogenic.

In addition, we also detected two type II variations of *CTNNB1* (*beta-catenin*) and *PIK3CA* genes, as well as six type III variations of *CREBBP*, *ERCC5, FANCI, FANCM, FAP*, and *PTPRT* genes.

The gene *CTNNB1* encodes beta-catenin protein, which acts as an intracellular signal converter in the Wnt signaling pathway. Aberrant *CTNNB1* activates proto oncogenes and cyclins, driving the occurrence, progress, survival, and recurrence of cancer. *CTNNB1* is recurrently mutated in multiple cancer types (19). According to the annotation in the OncoKB database, *CTNNB1* p.S37P mutation may be pathogenic.

*PIK3CA* gene encodes the catalytic subunit of class I phosphatidylinositol-3-kinase (PI3K). It plays an important role in the cell growth, proliferation, migration and survival through the PI3K/AKT/mTOR signaling pathway. Mutated *PIK3CA* gene leads to the continuously activated PI3K/Akt signaling pathway in various cancers (20–23). p.M1043I is located in the PI3K/PI4K domain of the PIK3CA protein. According to the annotation in the OncoKB database, *PIK3CA* p.M1043I alteration is an oncogenic variation, which can increase the level of AKT phosphorylation and activate the downstream signaling pathway (24, 25).

*CREBBP* is a tumor suppressor and transcriptional co-activator, and it is frequently inactivated in hematologic malignancies (26, 27). The association between *CREBBP* and carcinoma has not yet been reported. MutationTaster, SIFT and Polyphen-2 consistently predicted that *CREBBP* p.A1830T is harmful.

*ERCC5* is also a tumor suppressor and serves as a DNA endonuclease involved in the nucleotide-excision repair (NER) pathway (28). Germline mutations of *ERCC5* gene are associated with several disorders with defective DNA repair, which are susceptible to develop certain cancers (29). Although abnormal expression of ERCC5 has been detected in breast and ovarian cancers (30), somatic mutations of this gene have not yet been reported to be oncogenic drivers. *ERCC5* p.S1094L is a novel variation. MutationTaster, SIFT and Polyphen-2 predicted this mutation is disease causing, tolerated, and possibly damaging, respectively.

*FANCI* is an important component of the Fanconi anemia (FA) pathway, which is a DNA damage response (DDR) pathway (31). Among the identified 22 FA proteins (FANCA-FANCW), FANCI is an evolutionarily relevant partner of FANCD2. These two proteins form a protein complex FANCI-FANCD2 (ID2), which is a critical step in the activation of the FA pathway (32). A study showed that mutated *FANCI* may be a candidate ovarian cancer-predisposing gene (33). The non-frameshift deletion (p.A727del) in *FANCI* is a novel alteration, which is predicted to be disease causing by MutationTaster software.

*FANCM* is another essential member of the FA pathway. It is a tumor suppressor gene encoding a conserved and structure-specific DNA translocase. Loss-of-function mutations in *FANCM* are associated with predisposition to breast and ovarian cancer (34, 35). *FANCM* p.D879Y is a novel mutation. MutationTaster predicted that this variation is a polymorphism, whereas SIFT and Polyphen-2 predicted that it is deleterious and possibly damaging, respectively.

Fibroblast activation protein alpha (FAP) is a type II integral serine protease expressed specifically by activated fibroblasts. *FAP* can promote tumor growth, invasion, metastasis, and immunosuppression (36). MutationTaster, SIFT and Polyphen-2 predicted that the *FAP* p.E200K variation is disease causing, tolerant, and possibly damaging, respectively.

The *PTPRT* gene encodes a receptor-type tyrosine-protein phosphatase T enzyme. It is a tumor suppressor gene involved in signal transduction and cellular adhesion. *PTPRT* inhibits cell proliferation through the STAT3 pathway (37–39). MutationTaster, SIFT, and Polyphen-2 predicted that the *PTPRT* p.R461Q variation is disease causing, tolerant, and probably damaging, respectively.

Meanwhile, we identified four gene fusions through sequencing RNA samples, i.e., *LINC01138-NOTCH2, YWHAE-CRK, IGF2BP2-ETV5*, and *IGF2BP2-ETV5*. The association between these four gene fusions and malignancy has not yet been reported.

Regretfully, we didn’t detect the SMARCA4 (BRG1) deletion by NGS. The possible reason is that NGS can accurately detect small insertion/deletion mutations, point mutations and exonic copy-number changes. However, NGS cannot accurately detect large deletions. We speculate that the patient harbors a large SMARCA4 (BRG1) deletion. In addition, the alterations may occur in the regulatory regions of the SMARCA4 gene, such as the promoter region, CpG islands, enhancers, and so on, resulting in a loss of gene expression. Epigenetic modifications involving chemical alterations to chromatin or DNA molecules may also contribute to gene expression deficiency. These modifications include DNA methylation, histone modifications, and non-coding RNAs. For instance, DNA methylation refers to adding methyl groups to DNA molecules, often leading to gene silencing and suppressed expression.

Based on the above genetic analysis, we speculate that, in addition to *SMARCA4 (BRG1)* deficiency, other genetic alterations might also participate in the occurrence and progress of the undifferentiated carcinoma of the gallbladder in this patient, particularly the mutations in the *CTNNB1, KRAS, PIK3CA, TP53, CREBBP*, and *FANCI* genes (**Table 1**).

Conventional treatment is usually ineffective for *SMARCA4 (BRG1)*-deficient tumors. Based on the antagonism of SWI/SNF and polycomb repressive complex 2 (PRC2), SWI/SNF deletion leads to the loss of inhibition of EZH2 methyltransferase, which in turn results in PRC2-mediated tumorigenesis (40). Targeted therapy with EZH2 inhibitors might be effective (41). Clinical trials on *SMARCB1 (INI1)*-deficient rhabdoid tumors and epithelioid sarcomas have shown that EZH2 inhibitors have lasting anti-proliferative effects (42). In a phase II clinical trial of epithelioid sarcoma, 15% of patients achieved clinical remission, of which 67% achieved remission for at least six months (43). Therefore, the US Food and Drug Administration accelerated the approval of the first EZH2 inhibitor tazemetostat in January 2020 for treating locally advanced or metastatic epithelioid sarcoma patients. *In vitro* and *in vivo* studies have suggested that tazemetostat has the potential anti-tumor effects on *SMARCA4 (BRG1)*- and *SMARCA2 (BRM)*-deficient small-cell carcinomas of the ovary of hypercalcaemic type (SCCOHT) (44), which brings hope for the targeted therapy of other types of *SMARCA4 (BRG1)*-deficient undifferentiated tumors. In addition to EZH2 inhibitors, other possible targeted therapies include histone deacetylase inhibitors and DNA methyltransferase inhibitors. Furthermore, another potential therapy is immune checkpoint inhibitor (ICI) therapy. The patient gave up the therapy after discharge from the hospital because of personal reasons.

To the best of our knowledge, this is the first case of *SMARCA4 (BRG1)*-deficient undifferentiated carcinoma of gallbladder with genetic analysis. This case might contribute to the understanding of SWI/SNF-deficient carcinoma of gallbladder.

## Data availability statement

The sample data of this patient for this study is included in the article/**Supplementary Material**.

## Ethics statement

The work was approved by the Ethics Committee of Shengjing Hospital of China Medical University. The patients/participants provided their written informed consent to participate in this study. Written informed consent was obtained from the participant/patient(s) for the publication of this case report.

## Author contributions

XM: conceptualization. JM: postoperative care. TY: Collection data. NM: writing the draft. BN: Editing the manuscript. All authors contributed to the article and approved the submitted version.
